# Spatial Pattern of *Verticillium dahliae* Microsclerotia and Cotton Plants with Wilt Symptoms in Commercial Plantations

**DOI:** 10.1371/journal.pone.0132812

**Published:** 2015-07-13

**Authors:** Feng Wei, Wenjing Shang, Jiarong Yang, Xiaoping Hu, Xiangming Xu

**Affiliations:** 1 State Key Laboratory of Crop Stress Biology for Arid Areas, College of Plant Protection, Northwest A&F University, Yangling, China; 2 East Malling Research, New Road, East Malling, Kent, United Kingdom; USDA-ARS-SRRC, UNITED STATES

## Abstract

Spatial patterns of pathogen inoculum in field soils and the resulting patterns of disease may reflect the underlying mechanisms of pathogen dispersal. This knowledge can be used to design more efficient sampling schemes for assessing diseases. Spatial patterns of *Verticillium dahliae* microsclerotia were characterized in commercial cotton fields through quadrat and point sampling in 1994 and 2013, respectively. Furthermore, cotton plants with wilt symptoms, caused by *V*. *dahliae*, were assessed in six commercial cotton fields in 2013. Soil samples were assayed for the density of microsclerotia (expressed as CFU g^-1^ of soil) using a wet-sieving plating method and a real-time quantitative PCR method for the 1994 and 2013 study, respectively. The estimated inoculum threshold for causing wilt development on individual plants varied with the three fields: ca. 1.6 CFU g^-1^ of soil for one field, and 7.2 CFU g^-1^ of soil for the other two. Both quadrat and point sampling spatial analyses showed that aggregation of *V*. *dahliae* inoculum in soils was usually not detected beyond 1.0 m. Similarly, the spatial patterns of wilted cotton plants indicated that spatial aggregation of diseased plants were only observed below the scale of 1.0 m in six commercial cotton plantations. Therefore, spatial aggregation of both *V*. *dahliae* inoculum and cotton plants with wilt symptoms is not likely to be detected above the scale of 1.0 m for most commercial cotton plantations. When designing schemes for assessing wilt inoculum and wilt development, this scale needs to be taken into consideration.

## Introduction

Cotton (*Gossypium hirsutum* L.) is an economically important crop with a worldwide distribution. Verticillium wilt of cotton, caused by the soil-borne fungus *Verticillium dahliae* Kleb., is an important disease in most cotton-growing areas. Substantial yield losses and reductions in fibre quality can result from severe epidemics. The pathogen can survive for more than 10 years in the absence of hosts in soil as microsclerotia [1], which is the primary inoculum of cotton wilt. Reducing the density of viable microsclerotia in soil is one of the key methods to manage wilt. Usually, viable microsclerotium density in soil can be quantified prior to or at sowing in order to predict the risk of wilt [2–5]. A plating test with semi-selective media has been used for this purpose [2–5]. Recently, several molecular methods based on real-time quantitative PCR (qPCR) have been developed to quantify *V*. *dahliae* population densities in soil [6–10], with the detection sensitivities varying considerably among these methods. A high-copy-number target sequence has been used to increase sensitivity of qPCR detection for *V*. *dahliae* [7], and a relationship between copy number and density of microsclerotia (and the usual estimated CFU [colony forming unit] values via the plating method) has been established [10].

Spatial pattern of soil inoculum will affect sampling designs for assessing fungal inoculum and disease development. The scale at which aggregation of *V*. *dahliae* inoculum is detected varies greatly among studies, and even on the same crop species. For example, inoculum was aggregated at the scale ranging from 1 m × 1 m [11], to 15.2 m × 15.2 m in a potato field with a high level of wilt incidence [12], and intermediate scales between these two extremes [13]. Microsclerotia in cauliflower fields in California usually had a low degree of aggregation at the scale of 2 m × 2 m for all 12 sites and aggregation over a larger distance (> 2 m) was only observed for a few sites [14]. Aggregation of microsclerotia was detected within quadrat sizes less than 2 m × 2 m in a fallow field that had been previously planted to cotton [15]. Aggregation of mint plants with Verticillium wilt was evident within quadrats of size (0.76 m × 0.76 m) and there was more spread of the disease within than between rows [16]. In olives, aggregation of wilted trees were spatially aggregated for quadrat sizes up to 4 m × 4 m (i.e. 16 trees) [17].

With the recent advances in real-time PCR-based quantification of *V*. *dahliae* in soil [6–10], it is relatively easier to estimate inoculum density and to predict wilt risk prior to sowing, unlike the plating test which usually requires a minimum of eight weeks to complete. To design an efficient sampling scheme for estimating soil inoculum density, a better understanding of spatial aggregation of *V*. *dahliae* inoculum in soils is needed. The present study reports results on spatial patterns of *V*. *dahliae* inoculum and cotton plants with wilt symptoms in commercial plantations with varying levels of wilt incidence. Specifically, we assessed spatial patterns of (ii) soil inoculum determined from point and quadrat sampling at a range of scales (0.25–5.0 m), and (iii) cotton plants with wilt symptoms within and across rows.

## Materials and Methods

The density of *V*. *dahliae* microsclerotia was initially estimated using the wet-sieving plating method [18] for the quadrat sampling, and a new wet-sieving qPCR method [10] for the point sampling.

### Spatial pattern of microsclerotium density

In August 1994, three commercial cotton fields with different levels of wilt incidence (cv. Zhong12) in Yangling, Shaanxi Province, China were selected for quadrat sampling. In each field, a plot of 8.4 m × 8.4 m was marked out in the field centre; this plot was then divided into 49 contiguous quadrats of 1.2 m × 1.2 m (within each quadrat, there were two rows, each with 5–6 plants). Within each quadrat, a single core (ca. 2.5 cm in diameter) sample of soil (at depth 0–20 cm) was sampled in late August at each point: near the four corners and the quadrat centre; these five samples were then mixed together, resulting in a single composite sample for each quadrat. Soil inoculum density was estimated using the wet-sieving plating method [18]. These three fields were named as Y_A, Y_B and Y_C (S1 Table). Disease incidence (0 to 100%) was estimated at the same times for each field plot, consisting of 500–550 plants by counting the number of plants with foliar symptoms of Verticillium wilt (S1 Fig).

**Fig 1:**
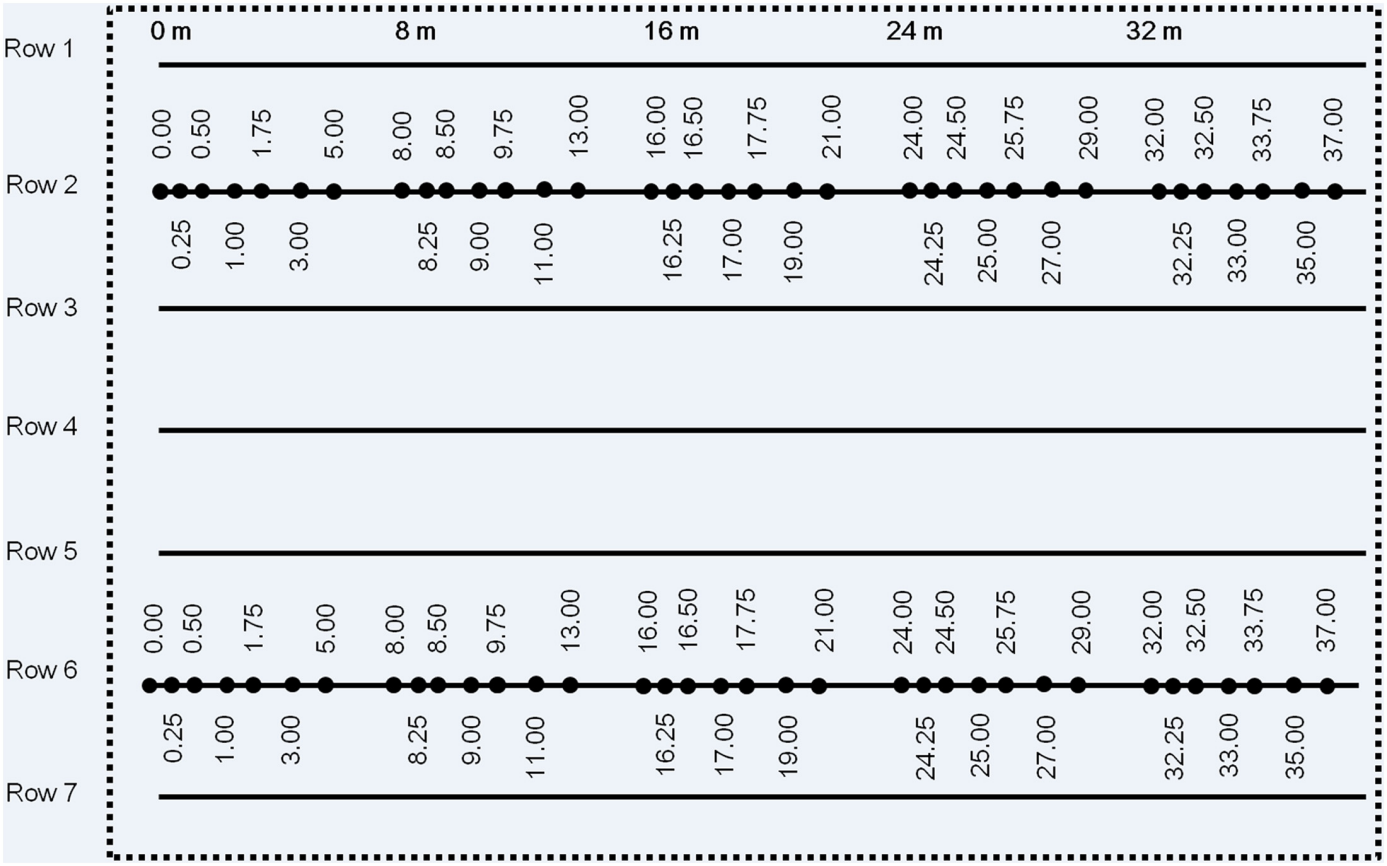
A diagram illustrating the point sample positions. Soil was sampled in three commercial cotton fields to study spatial relationship in the density of *Verticillium dahliae* inoculum. Numbers indicate the distances (m) from sampling points to first sampling point in the same row. Sampling was conducted in three commercial fields in late August 2013, Weinan, Shaanxi Province, China.

Point sampling was carried out in 2013 to investigate spatial pattern of microsclerotium density at shorter distances (< 1.2 m) than the quadrat sampling in 1994. Soil samples were taken from three randomly selected commercial cotton fields (oral permitted by the field owner, Jindong Song, Junyao Zhao, and Youpeng Shao, respectively) in Weinan, Shaanxi Province, China in August 2013 (S2 Table). These three fields (W_A, W_B and W_C) were in the traditionally cotton production area but cotton production in the area has been decreasing significantly during the last two decades. Cultivar identity in the three fields was unknown. Plants were grown in two rows in each raised bed (width 60 cm), with 40 cm between the two rows and 80 cm between neighbouring bed centres (giving across-row distance of ca. 40 cm); within-row plant-to-plant distance was ca. 30 cm. Within each field, a plot with 7 consecutive rows of 40 m long was randomly selected (with one constraint—the plots were at least 5 m away from the field edges). In row 2 and 6, soil was sampled in five clusters with an inter-cluster distance of 8.0 m; within each cluster seven individual soil samples were taken at the positions of 0.00, 0.25, 0.50, 1.00, 1.75, 3.00 and 5.00 m (Fig 1). A single sample consisted of a single core (ca. 2.5 cm in diameter) at the depth of 20 cm. Inoculum density of *V*. *dahliae* in each sample was estimated using a qPCR method [10].

### Estimation of soil inoculum via the wet-sieving plating

The density of *V*. *dahliae* inoculum in each soil sample was estimated using a well-established wet-sieving and plating method [18]. Soil samples were first air-dried for 3 weeks to kill conidia and the mycelial fragments of *V*. *dahliae* before sieving. A sample of 20 g soil was placed into a screw-cap bottle and distilled water was added to a volume of 100 ml. The bottle was shaken vigorously for 1 h on a reciprocating shaker at 175 rpm to break-down soil clumps. Then the suspension was washed through 20 μm sieves with 20 cm in diameter, and the residues on the 20 μm sieve (Dahengqiao Laboratory Instrument Ltd., Zhejiang, China) were collected and dispensed into the original bottle, distilled water was added to a volume of 100 ml; the resulting suspension was shaken thoroughly and then 2 ml of the soil suspension were transferred with a pipette to each of 10 modified soil extract agar (MSEA) plates (90 mm in diameter). A glass spreader was used to distribute the 2 ml suspension over the MSEA plate surface. The plates were then incubated at 22°C for 4 weeks in the dark, after which the soil was washed away from the surface and the plates were then inverted to dry at room temperature, and then scanned for colonies of *V*. *dahliae* at 24× under a dissecting microscope (SMZ-10 Nikon, Japan). The sieves were thoroughly washed and rinsed between samples by means of an ultrasonic cleaning bath.

### Estimation of soil inoculum via a wet-sieving qPCR method

This wet-sieving qPCR method was recently developed that can quantify *V*. *dahliae* soil inoculum as low as 0.5 CFU g^-1^ of soil, and the estimated CFU is linearly highly related to the CFU values estimated by the wet-sieving plating method (R^2^ = 0.96) [10]. Initially, an air-dried soil sample of 20 g was treated as in the wet-sieving plating method. The residual contents on the 20 μm sieves after washing were washed into a 50 ml centrifuge tube. The tube was then centrifuged at 8,000 rpm for 5 min. The supernatant was discarded. The left pellet was kept at -60°C for 12 h in a vacuum freeze dryer (SIM, USA). All the dried pellets were weighted and a tenth of weight (0.20–0.25 g) was used for DNA extraction. Each sample was placed in a 2 ml DNA extraction tube containing lysing matrix E, which is a mixture of ceramic and silica particles to lyse all soil organisms efficiently, frozen in liquid nitrogen for 1 min, and then placed in a Micro Homogenizing system (Tomy MS-100, Japan) at 5000 rpm for 40 s. These two steps were repeated before adding of 978 μl of sodium phosphate buffer, and 122 μl of MT buffer (supplied with the FastDNA Spin kit). Total DNA of soil samples were extracted using FastDNA Spin Kit for Soil (MP Bio-medical, USA) following the manufacturer′s protocol with several modifications as follows: (i) instead of allowing the Binding Matrix to settle, the tubes were briefly centrifuged at 11,500 rpm and then the supernatant was removed, (ii) the pellet was washed by gentle re-suspension in 1 ml of 5.5 M guanidine thiocyanate (Sigma, USA) and tubes were centrifuged briefly at 11,500 rpm; then the supernatant was removed until the silica had returned to its original color, (iii) after the last wash, the silica was re-suspended in 1 ml of 5.5 M guanidine thiocyanate before being transferred to a spin filter for centrifugation. To ensure comparability between samples, the final elution volume of 50 μl was used for all samples and 1 μl DNA solution was used as template for qPCR assay.

A primer pair specific to *V*. *dahliae* (forward Vd-F929-947: 5′-CGTTTCCCGTTACTCTTCT-3′, and reverse Vd-R1076-1094: 5′-GGATTTCGGCCCAGAAACT-3′) was designed from the high copy number intergenic spacer (IGS) of rDNA [7]. Amplification reactions were performed in 8-well tubes using an iQ5 ICycler instrument (Bio-Rad Laboratories, CA, USA). PCR reactions of 25 μl contained 1 μl of template DNA, and 12.5 μl 2×UltraSYBR Mixture with Rox (ComWin Biotech, Beijing, China), 1 μM of each of the primer pair, and 10 μl of nanopure water. The amplification program consisted of initial denaturation of 10 min at 95°C followed by up to 40 cycles of 95°C for 30 s, 62°C for 30 s and 72°C for 30 s.

A series of samples with known copy numbers of *V*. *dahliae* IGS in plasmid were used to construct a standard curve for qPCR. The *V*. *dahilae*-specific band amplified by the primer pair was purified using an Agarose Gel Extraction Kit (Roche Inc., Germany) and cloned into the pMD18-T vector (TaKaRa Biotechnology, Japan). The recombined vector was used to transform *Escherichia coli* DH5α competent cells using the electro-transformation method [19]. The number of copies of DNA μl^-1^ was estimated using a standard curve [10]. A 10-fold serial dilution of the plasmid DNA, ranging from 3.25×10^0^ to 3.25×10^5^ copies μl^-1^ (total six concentrations), was prepared and stored at -40°C until use for constructing a standard curve in duplicate for every plate (i.e. every qPCR run). Sterilised nanopure water was used as a negative control. All samples in this study were amplified in quadruplicate. Thus, a new standard curve relating Cq (quantification cycle) to DNA copy number was estimated for each run and used to estimate DNA copy number for each sample. The estimated DNA copy number by qPCR for each sample was then converted into microsclerotium number based on a previously established relationship between copy number and number of microsclerotia [10]. Namely, soil samples (from soil grown with non-*Verticillium* hosts for many years—hence likely to be free of *V*. *dahliae* inoculum; the soil was also autoclaved at 121°C and 115 kPa twice for 45 min each to kill microorganisms) were amended with known number of *V*. *dahliae* microsclerotia to obtain soil samples with one of the following nine microsclerotium densities: 0, 0.5, 1, 5, 10, 50, 100, 200, 500 and 1000 microsclerotia per gram of dry soil. Three replicates for each inoculum density were analysed using the qPCR method. A linear model (*y* = 48.429 *x*) was then derived to relate the estimated DNA copy numbers (*y*) to the corresponding microsclerotium density (*x*) [10].

### Spatial pattern of cotton plants with wilt symptoms

Wilt development was assessed in six commercial fields: the three fields used for point sampling of soil inoculum (W_A, W_B and W_C) (S3 Table) and three fields in the Xinjiang Autonomous Region, China during the period of late August to early September 2013 (S4 Table). Cotton in Xinjiang was normally grown in larger state farms, receiving far less management interventions than in Weinan where W_A, W_B and W_C were located. The distance between the two sampling areas was > 2500 km. Three fields in Xinjiang (X_A, X_B and X_C) were selected for wilt assessment, representing each of the three levels of wilt development (low, moderate and severe).

In W_A, W_B, and W_C, wilt on all plants was recorded for the seven rows for a length of 40 meters (Fig 1) (soils in row 2 and 6 in each field were used for point sampling to study the spatial relationship of inoculum density); there were ca.140-150 plants per row within the length of 40 m. In Xinjiang, wilt was assessed on all plants of cv. Xinluzhong28 (tolerant to wilt) in a plot of size 20 rows with 300 plants per row in each field. Plants were grown in double rows (distance ca. 10 cm); every six rows (i.e. three double rows) were cover under the same polythene. There was a gap of ca. 60 cm between two ‘double-rows’ where a pipeline was located to provide dripping irrigation—two gaps among the six consecutive rows. There was a distance of 20 cm (instead of 60 cm as there was no pipeline) between the two ‘six-rows’ units. Within each row, the plant-to-plant distance was ca. 10 cm. Wilt severity on individual plants based on foliar symptoms (S1 Fig) was recorded on a scale of 0 to 4: 0—no symptoms; 1—≤ 25% leaves with wilt symptoms; 2—leaves with wilt symptoms > 25% and ≤ 50%; 3—leaves with wilt symptoms > 50% and ≤ 75%; and 4—> 75% leaves with wilt symptoms [20]. In all fields, locations of gaps (missing plants) were also recorded.

### Data analysis

The qPCR data were analysed with the iQ5 software (version 2.1) to generate amplification, standard, and melting curves. Amplification efficiency (E) was calculated as $E=10^{-slope^{-1}}-1$, where slope is the slope of the standard curve using dilution series plamids of intergenic spacer (IGS) fragment.

For the quadrat data, aggregation of *V*. *dahliae* inoculum within quadrats was tested by first assessing whether CFU values of individual quadrats could fit a Poisson distribution and then calculating spatial autocorrelation up to a spatial lag distance of 4.8 m (i.e. over four consecutive quadrats). Counts data of microsclerotia are expected to follow a Poisson distribution if individual microsclerotium was randomly distributed at the scale of assessment. If CFU values failed to fit a Poisson distribution, the data were fitted to a negative binomial distribution.

For the point data, CFU data were first transformed to the natural logarithm. Row effects were removed within each field by subtracting the row average CFU from the individual CFU values. Spatial autocorrelation up to a spatial lag of 16 m was then calculated for the data pooled over the two rows in each field. The number of pairs of data points at a given spatial lag was recorded in order to estimate the significance of an observed spatial autocorrelation.

Join-count statistics were used to determine whether cotton plants with wilt symptoms were spatially aggregated and, if so, the spatial scale at which the aggregation was observed. The join-count analysis is closely related to the autocorrelation for binary data (diseased or healthy). In join-count analysis, individual plants were classified either as diseased (D) when wilt score > 0 or healthy (H). Join-count for pairs of plants at a certain distance (i.e. spatial lag, quantified as number of plants) apart with the same status (i.e. HH or DD) was calculated from a given data set (wilt status of all plants assessed in a given field). Join-count statistics (i.e. number of HH or DD pairs) were calculated for both within and across rows for pairs of plants with a distance up to 20 plants apart. Join-count statistics were obtained for individual rows within each field and then pooled over all within-rows for each field. Across-row join-count analysis was only applied to three fields in Weinan (W_A, W_B and W_C) because of unequal row-to-row distances at individual fields in Xinjiang (X_A, X_B and X_C).

Instead of using parametric tests of join-count statistics [21] for existence of significant association, permutation test was used to assess whether join-count statistics (DD) for the observed data was greater than those obtained under the assumption of a random pattern. Permutation test was used because there was varying number of missing plants within each sampling plot. In each permutation, each of observed wilt score within each row was randomly assigned to a plant at a non-missing position in the same row; thus locations of the gaps [i.e. positions with missing plants] in the assessed rows were never allowed to be assigned wilt scores. Therefore, the permutation was conditional on those positions where plants were present at the time of assessment. For each permutated data set, join-count statistics were obtained as for the observed data set. A total of 199 permutations were done for within-row or across-row joint-count tests for each field. Therefore, there were 200 (199 permutated ones and one observed) DD values at each distance lag for a given direction (within or across row) in each field. Significance of DD at each lag was determined by comparing the observed value with the corresponding 199 values from permutated data sets.

To study the relationship of the inoculum density with wilt severity on individual plants, wilt severity score data were first converted to two outcomes (healthy or wilt), because only a few plants had wilt scores of 2 or above, particularly for fields W_B and W_C. A logistic model was then fitted to this new data set assuming that errors follow a binomial distribution, i.e. ln[*p*/(1-*p*)] = *α*+*βx*, where *x* is the transformed CFU value: *x* = log_10_(*CFU*+1). An inoculum threshold for causing wilt development on individual plants was then estimated as *x* = 10^-α/β^-1 (i.e. when the probability of wilt development is 0.5). The three fields were treated as a factor when fitting the equation.

Autocorrelation and join-count statistics (including permutation analysis) were calculated using programming codes specifically written in R (version 3.0.3).

## Results

### Spatial pattern of *V*. *dahliae* microsclerotia in soil

**Fig 2:**
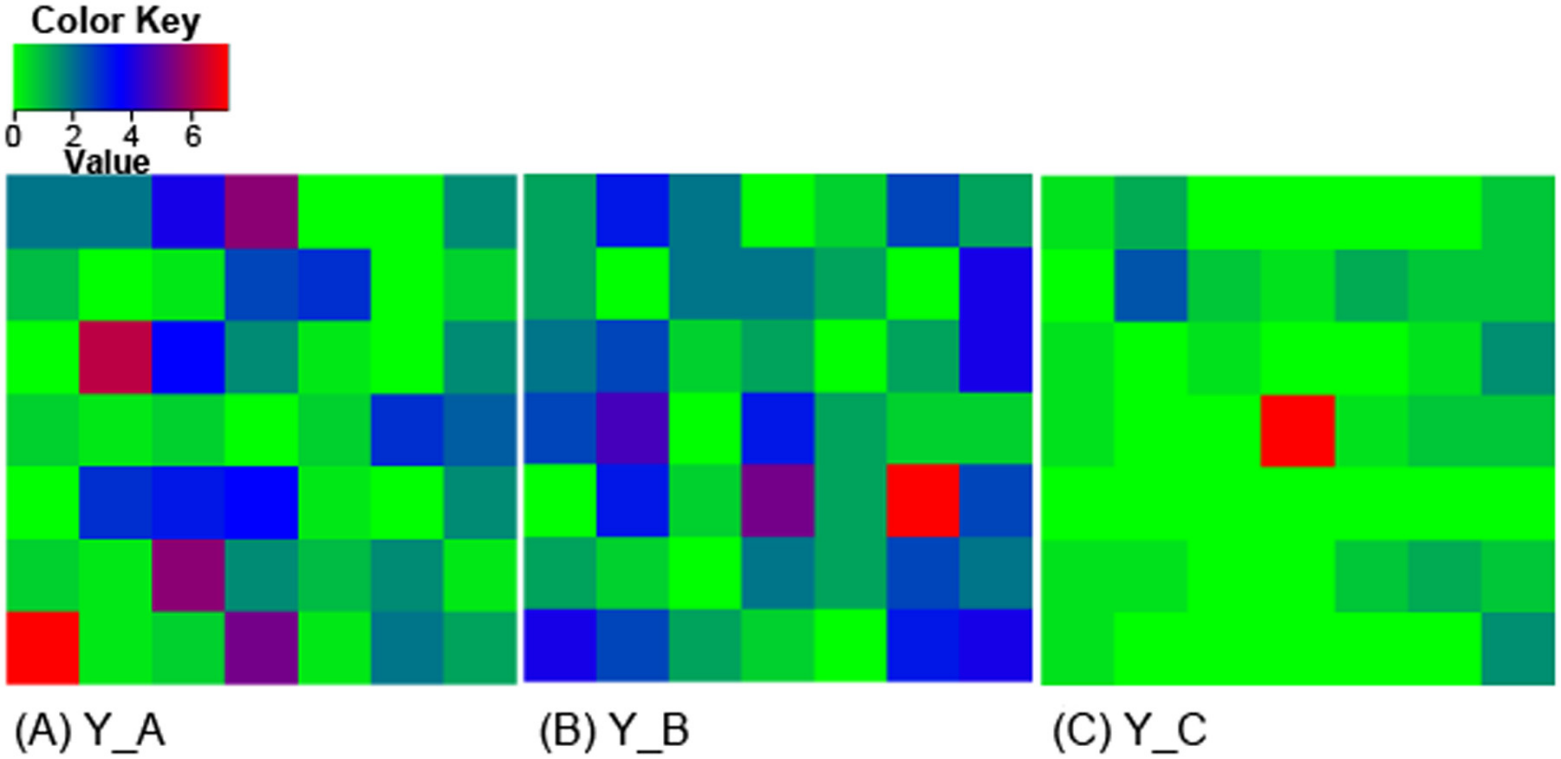
Spatial pattern of *Verticillium dahliae* inoculum in quadrats. The inoculum density (CFU g^-1^ of soil) of *V*. *dahliae* were estimated in three commercial cotton fields (Y_A, Y_B, and Y_C) using the wet-sieving plating method. Within each field, the sampling plot was divided into 49 (7 × 7) quadrats of the 1.2 m × 1.2 m size. Within each quadrat, a single core (ca. 2.5 cm in diameter) sample of soil (at depth 0–20 cm) was sampled in late August at each of five points: near the four corners and the quadrat centre point; these five samples were then mixed together, resulting in a single composite sample for each quadrat.

The incidence of wilt in the three fields used for quadrat sampling of microsclerotia was 46%, 38% and 14% for field Y_A, Y_B and Y_C, respectively. Spatial distribution of inoculum in the 49 contiguous quadrats is shown in Fig 2 for the three fields. Y_A and Y_C had an overall greatest and lowest CFU, respectively. CFU values per gram of soil ranged from 0 to 9 (mean = 2.08, variance = 5.06) for Y_A, from 0 to 5 (mean = 1.15, variance = 0.91) for Y_B, and from 0 to 8 (mean = 0.55, variance = 1.23) for Y_C. Of the three fields, only CFU data from Y_A could not be fitted to a Poisson distribution but were satisfactorily described by a negative binomial distribution (estimated parameter k value = 0.954 ± 0.340). Spatial autocorrelation was not statistically significant for all spatial lags (up to 4.8 m) for any of the three fields: all spatial autocorrelation coefficients were within the range of -0.22 and 0.26.

**Fig 3:**
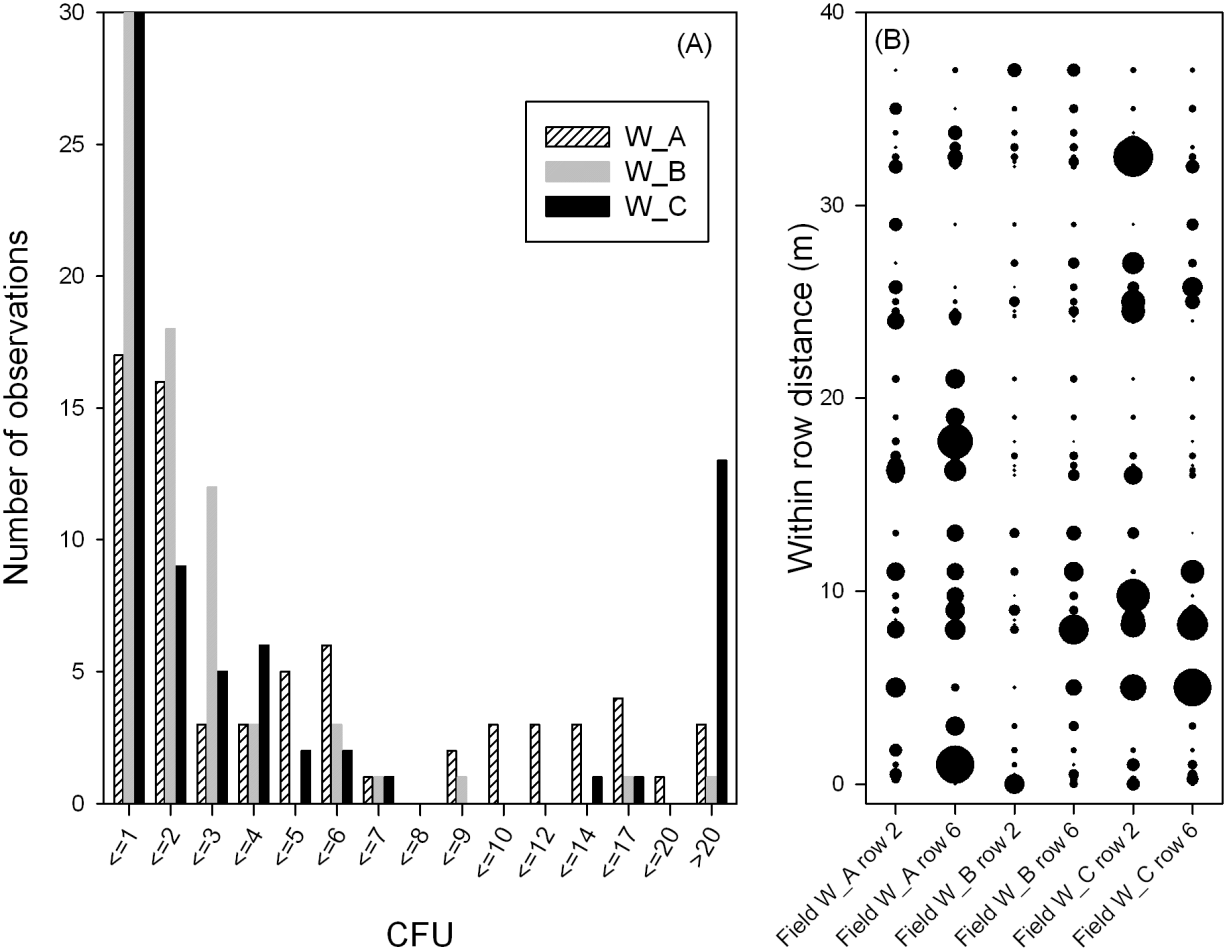
Inoculum density of *Verticillium dahliae* from point sampling. Histogram (A) and spatial pattern (B) of estimated inoculum density (CFU g^-1^ of soil) of *Verticillium dahliae* in three commercial cotton fields (W_A, W_B, and W_C) using a real-time quantitative PCR method. Within each row, 35 soil samples (at depth 0–20cm) were taken; the circle size is proportional to the CFU estimate at the point (the largest circle corresponds to 300 CFU g^-1^ of soil in W_C).

A total of 11 qPCR runs were carried out to quantify *V*. *dahliae* inoculum density for 210 soil samples from W_A, W_B and W_C fields. In every run, there was a linear relationship (R^2^ > 0.98 with an average of 0.994) between the log of copy number of the *V*. *dahliae* IGS fragment and Cq (quantification cycle) value; an example of such a relationship is shown in Fig 3. The amplification efficiency ranged from 0.96 to 1.08 with an average of 1.04. The Cq values for no template controls (NTC) were > 40 or no amplification was observed.

**Fig 4:**
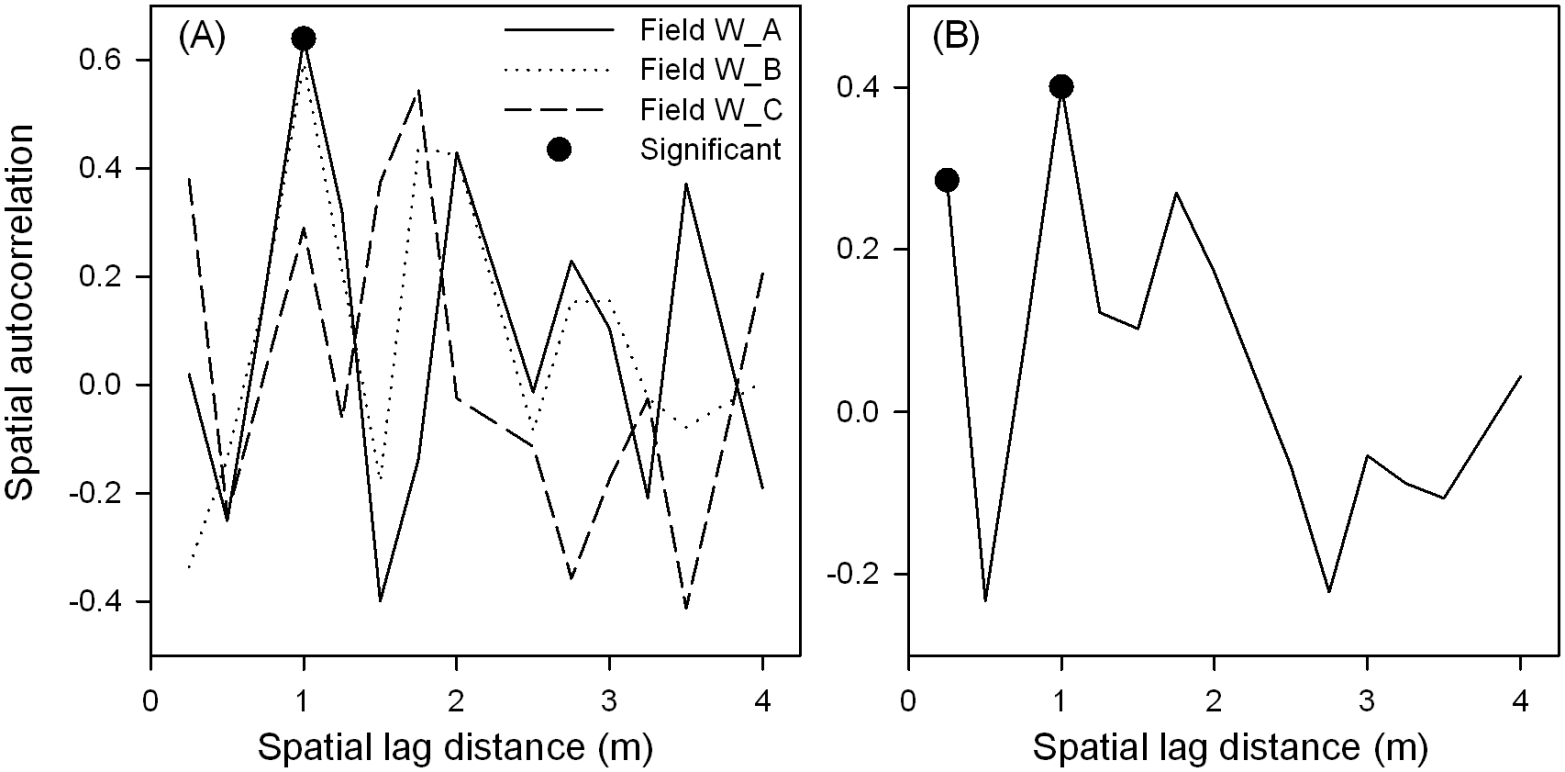
Spatial autocorrelation of inoculum density of *Verticillium dahliae* from point sampling. (A) individual fields, and (B) pooled over three fields. Within each field, 70 samples were collected by point sampling (at depth 0–20 cm).

The distribution of estimated CFU values was skewed for the three fields (Fig 3A), particularly for W_B and W_C. In W_B, there were more samples with low CFU values (≤ 3.0), whereas in W_C, there were more samples with either low (≤ 1.0) or high (> 20) CFU values (Fig 4A). There were large variations among estimated CFU values within each field (Fig 3). CFU values per gram of soil ranged from 0.0 to 249.0 (median = 2.5) for W_A, from 0.0 to 73.0 (median = 1.3) for W_B, and from 0.0 to 300.4 (median = 1.5) for W_C. Only 17 out of the 210 samples had inoculum density more than 20.0 CFU g^-1^ of soil: 13 of these samples were from W_C (Fig 3A). Only seven samples had CFU values > 50 g^-1^ of soil: 73.2, 90.6, 124.0, 153.4, 224.7, 249.3 and 300.4.

When spatial autocorrelation was calculated separately for each field, only correlation at the spatial lag of 1.0 m was statistically significant (P < 0.05, Fig 4A) in field W_A. When pooled over all three fields, autocorrelation was significant (P < 0.05) only at spatial lags of 0.25 and 1.0 m (Fig 4B).

### Spatial pattern of cotton plants with wilt symptoms

**Fig 5:**
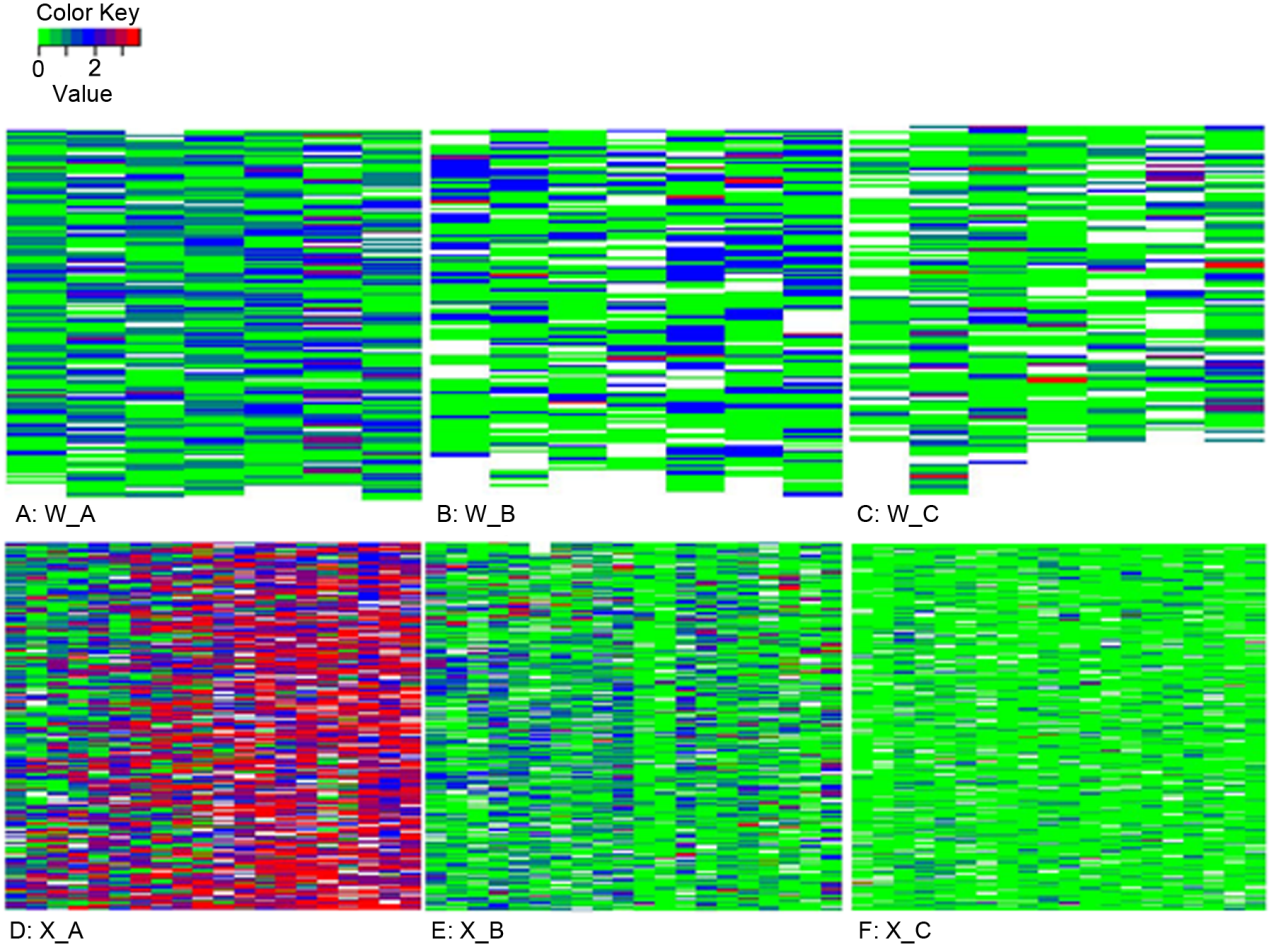
Spatial pattern of wilted cotton plants in commercial fields. Wilt severity was assessed in late August and early September on a scale of 0–4: 0—no symptoms; 1—leaves with wilt symptoms ≤ 25%; 2—leaves with wilt symptoms > 25% and ≤ 50%; 3—leaves with wilt symptoms > 50% and ≤ 75%; and 4—>75% leaves with wilt symptoms. Although wilt incidences for W_B and W_C were similar (33% vs. 34%), the apparent difference in the heat maps (B-C) was likely due to the differences in number of missing plants in each field: 15% and 23% of plants were missing for W_B and W_C, respectively. White colour indicates a missing plant position.

Fig 5 shows wilt severity for W_A, W_B and W_C; wilt incidence was 56%, 33% and 34% for field W_A, W_B and W_C (Table 1), respectively. For all three fields, join-count statistics were significant (P < 0.01) for within-row neighbouring plants (Table 2). The DD values for within-row neighbouring plants (i.e. lag 1, 0.3 m) were 588, 503 and 337 for W_A, W_B and W_C, respectively, compared to the corresponding maximum values of 491, 447 and 319 from the 199 permutations. The join-count value at within-row lag 2 (0.6 m) was significant at 5% for W_B only. None of the across-row join-count statistics was statistically different from the expected under the assumption of a random pattern.

**Table 1:** Number of cotton plants in each wilt category, assessed in six commercial plantations in 2013.

Field^a^	Wilt severity score^b^				
	0	1	2	3	4
W_A	429	354	148	34	1
W_B	558	268	12	0	0
W_C	419	139	46	21	7
X_A	825	712	853	1513	1585
X_B	3121	1485	589	177	105
X_C	4704	730	66	13	10

^a^ Three plantations were located in Weinan, Shaanxi (W_A, W_B, W_C) and three in Xinjiang (X_A, X_B, X_C).

^b^Wilt severity on individual plants was recorded on a scale of 0 to 4: 0—no symptoms; 1—≤ 25% leaves with wilt symptoms; 2—leaves with wilt symptoms > 25% and ≤ 50%; 3—leaves with wilt symptoms > 50% and ≤ 75%; and 4—> 75% leaves with wilt symptoms.

**Table 2:** Within-row join-count test results for aggregation of cotton plants with wilt symptoms in three commercial fields in Weinan, Shaanxi for spatial lags with significant or close-to-significant aggregation detected. Wilt was assessed in late August 2013. There was no between-row aggregation for three fields in Weinan; between-row join-count analysis was not done for the three Xinjiang fields because of variable row-to-row distances.^a^

**Weinan**	**Spatial lag (distance in meters)**			
	0.3	0.6	0.9	
W_A	**588**	471	407	
	(413, 491, 1)	(415, 500, 36)	(415, 494, 200)	
W_B	**503**	**424**	415	
	(382, 447, 1)	(369, 437, 8)	(367, 427, 134)	
W_C	**337**	280	252	
	(268, 319, 1)	(255, 309, 99)	(245, 309, 198)	
**Xinjiang**	**Spatial lag (distance in meters)**			
	0.1	0.2	0.3	0.4
X_A	**4056**	**3953**	**3886**	**3863**
	(3814, 3912, 1)	(3778, 3885, 1)	(3759, 3885,1)	(3745, 3889, 3)
X_B	**2998**	**2830**	**2791**	2744
	(2671, 2854, 1)	(2652, 2818, 1)	(2651, 2846, 2)	(2609, 2802, 29)
X_C	**3929**	3794	3265	3789
	(3789, 3903, 1)	(3770, 3857 169)	(3747, 3850, 186)	(3732, 3829, 67)

^a^Data in bold figure indicate a significant join-count DD value (i.e. aggregation of wilted plants); the three numbers in the bracket represent the minimum and maximum of the join-count DD values in the 199 permutations, and the rank (in the descending order) of the observed join-count in the 200 values (199 permutations and the observed), respectively. The probability of aggregation at a specific lag is estimated by dividing the rank by 200.

Incidence of wilt was much higher in field X_A (85%) than in field X_B (43%) and X_C (15%) (Table 1); furthermore, wilt was more severe in X_A than X_B and X_C (Fig 5D–5F, Table 1). About 56% of plants in X_A had wilt scores of at least 3, compared to 5% in X_B and 0.4% in X_C. Within-row join-count statistics (DD) were significantly greater than the expected under the assumption of a random pattern for lag 1–3 (0.1–0.3 m, P < 0.01) and lag 4 (0.4 m, P < 0.05) for X_A (Table 2). For X_B, within-row join-count statistics were significant for lag 1–3 (0.1–0.3 m, P < 0.01) (Table 2). For X_C, within-row join-count statistics were only significant (P < 0.01) for neighbouring plants, i.e. lag 1 (0.1 m) (Table 2).

### Relationship between CFU and wilt status of individual plants

**Fig 6:**
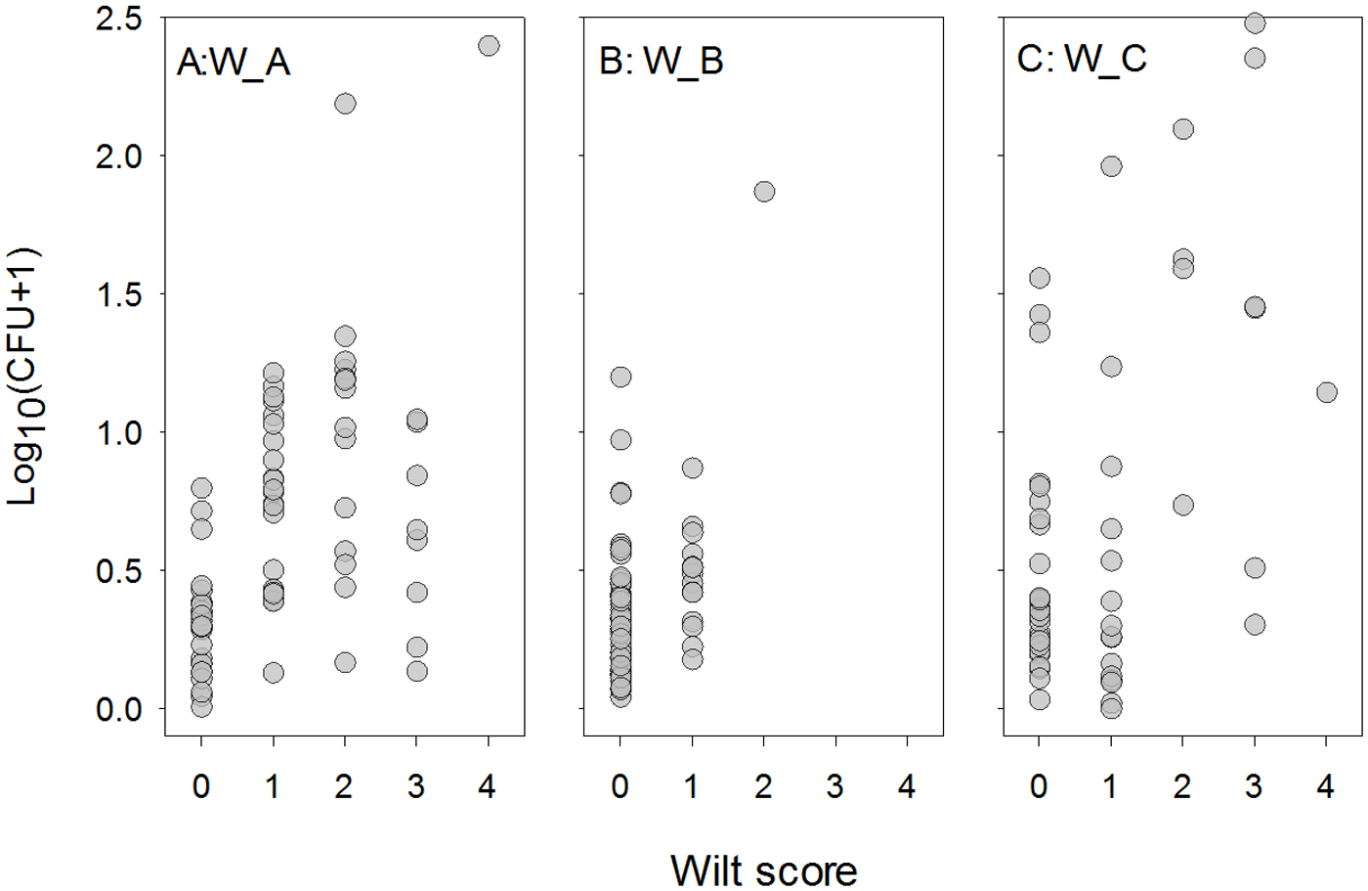
Estimated *Verticillium dahliae* inoculum densities in soil plotted against wilt scores of individual plants. These plantations (W_A, W_B, W_C) were located in Weinan, Shaanxi. The distance between soil sampling points and the plant stem was ca. 15 cm. Wilt on each plant in each plant was scored as: 0—no symptoms; 1—leaves with wilt symptoms ≤ 25%; 2—leaves with wilt symptoms > 25% and ≤ 50%; 3—leaves with wilt symptoms > 50% and ≤ 75%; and 4—>75% leaves with wilt symptoms.

Increasing CFU values in general led to higher incidence (Fig 6). This relationship can be satisfactorily described by a logistic model:

for the field W_A: ln[*p*/(1-*p*)] = -1.582+4.378*x*

and for the fields W_B and W_C: ln[*p*/(1-*p*)] = -1.582+1.729*x*.

The standard errors for the three parameter estimates were 0.300 (the intercept), 0.804 (the slope for W_A) and 0.470 (the slope for W_B and W_C). This model explained about 23.6% of the total deviance. Based on the models, threshold CFU values for wilt infection were estimated to be ~1.6 and 7.2 g^-1^ of soil for the field W_A and W_B/W_C, respectively.

## Discussion

The spatial aggregation of *V*. *dahliae* microsclerotia and plants with wilt symptoms is most likely within a distance of 1.0 m in commercial cotton fields in China. The distribution of microsclerotium density was skewed for all testing fields. There were large variations among estimated CFU values within each field. For cotton plants with wilt, join-count statistics were significant for within-row neighbouring plants.

Quantitative real-time PCR is now commonly used for rapid and accurate quantification of fungal biomass. This approach relies on a standard curve relating Cq values to the actual copy number of particular DNA target [22, 23]. The present study, unlike previous studies on quantification of *V*. *dahliae* DNA [7–9], used a cloned target sequence as the standards in all qPCR runs to establish the standard curves. A cloned target DNA sequence is more stable than PCR products or genomic DNA [22]. All the qPCR runs had amplification efficiency within 0.96–1.08. The relationship between IGS copy and microsclerotium numbers may vary with individual *V*. *dahliae* strains since there are about 24–73 copies (average of ≈46) of this region per haploid genome of *V*. *dahliae* [7]. This variability is relatively small when compared to the variability in the diameter of microsclerotia in naturally infested soils: ranging from 11 to 125 μm [24]. Indeed given our limited knowledge on the germination behaviour of individual microsclerotia (e.g., repeated germination, which could be related to the microsclerotium size), we are unlikely to be able to estimate the true inoculum potential of *V*. *dahliae* microsclerotia; this also applies to the standard wet-sieving method. Inhibition of PCR amplification in soil may also cause errors in inoculum estimation. The soil DNA extraction method used in this study was able to reduce much the inhibition of PCR [10], which used soil samples from the same three fields (W_A, W_B, and W_C) as the current study.

The estimated inoculum densities for the three fields in Yangling (0–9 CFU g^-1^ of soil) were lower than the three fields in Weinan (0–300 CFU g^-1^ of soil: 177 of the total 184 samples had CFU < 50). For the Yangling fields, soils were sampled using a quadrat sampling scheme and CFU quantified by the wet-sieving plating method. On the other hand, for the Weinan fields soils were sampled as composite samples of five cores and CFU quantified by a wet-sieving qPCR method. These two CFU quantification methods produced comparable levels of estimated inoculum density [10], which are similar to those estimated CFU values in the present study. In addition to the differences in inoculum density between plots, the large differences in estimated inoculum densities between the Yangling and Weinan sites are most likely caused by the differences in sampling as a consequence of pooling five core soil samples. The overall incidence of Verticillium wilt increased with increasing inoculums density for a given site as demonstrated in previous studies [2–5, 10]. Moreover, the estimated inoculum threshold values for causing wilt on individual plants are similar to those values published previously [10], depending on cultivars.

Aggregation of *V*. *dahliae* inoculum in soil or cotton plants with wilt symptoms was not likely to be beyond the scale of 1.0 m. The extent of aggregation appeared to increase with increasing disease severity, which has been observed in many experimental and theoretical models [21, 25, 26]. The present finding of aggregation of *V*. *dahliae* inoculum in soil and wilted plants at a small scale agrees with findings of several previous studies on *V*. *dahliae* of other host plants. For example, aggregation of *Verticillium* inoculum in mint fields [16] and in cauliflower fields in California was usually around 2 m [14]. However, aggregation of *V*. *dahliae* inoculum was observed at a much larger scale (ca. 15 m) in potato fields [12, 13]. The nature of potato tuber harvesting may explain this result: soil can potentially be moved during potato harvesting, and not in cotton, where individual plants are usually pulled out from soil manually in China.

Inoculum density of *V*. *dahliae* in soil is known to vary within and between seasons. For example, inoculum density was lower in the spring than in the fall in potato fields [27, 28]. Although the density of microsclerotia decreased in cotton field soils during spring and summer, infested plant debris at harvest that was released to the soil in fall likely to increased inoculum density [29, 30]. Recently, there are also study demonstrating that inoculum density in soil in the spring, summer and even winter were higher than those recorded in September/October in cauliflower fields [31]. In the present study, we were primarily interested in the spatial correlation of inoculum density instead of the absolute inoculum potential. Thus, the seasonal dynamics in inoculum density (indeed possible errors in the qPCR method) is not unlikely to alter the conclusions regarding spatial correlation qualitatively.

As reported previously in mint fields [16] and olive orchard [17], more clustering was detected within than across crop rows, indicating more spread of the disease within than between rows in the three fields in Weinan. Disease spread within rows could result from (i) scattering of infected cotton debris at harvest, which is more likely to occur within rows, (ii) root contact between plants being more likely within rows than across rows, and/or (iii) dispersal of inoculum in irrigation water along rows. Microsclerotia of *V*. *dahliae* can be carried in water and were detected in irrigation water at the ends of, but not at the beginning of, irrigation furrows in potato crops [32], and in olive groves [33].

In summary, the present study showed that clustering of *V*. *dahliae* inoculum and plants with wilt symptoms is most likely within a distance of 1.0 m. Thus, to estimate the overall inoculum level of a given field for predicting wilt risk prior to planting, a simple random sampling plan with minimum spatial distance of 2.0 m between any two samples should be able to provide an unbiased estimate, whilst the number of samples determines the accuracy of the estimate. Such a random sampling plan can be further enhanced by stratification, taking into account other field-specific features that may affect inoculum production and survival of *V*. *dahliae*. Such features may include soil characteristics and the presence of moisture gradients. On the other hand, to study spatial dynamics of *V*. *dahliae* in cotton, minimal spatial distance between two samples needs to be much less than 1.0 m.

## Supporting Information

S1 Fig Symptoms of cotton Verticillium wilt caused by *Verticillium dahliae*. A whole cotton plant with severe wilt (A) and a cotton leaf with the typical Verticillium wilt (B). Photos were taken in a commercial cotton field.
(TIF)

S2 FigStandard curve of real time quantitative PCR (qPCR).A standard curve relating the quantification cycle value to copy number (logarithmically transformed) of dilution series plamids of intergenic spacer (IGS) fragment from the JY strain. Amplification efficiency was $E=10^{-slope^{-1}}-1$.(TIF)

S1 Table *Verticillium dahliae* inoculum densities in soil in Yangling fields (quatdrats sampling). (XLS)

S2 Table *Verticillium dahliae* inoculum densities in soil and wilt scores of individual plants in Weinan fields (point sampling). (XLS)

S3 Table Spatial patterns of wilted cotton plants in Weinan fields. (XLS)

S4 Table Spatial patterns of wilted cotton plants in Xinjiang fields. (XLS)

## References

[1] Pegg GF, Brady BL. Verticillium wilts. New York: CABI Publishing; 2002.

[2] Harris DC, Yang JR. The relationship between the amount of *Verticillium dahliae* in soil and the incidence of strawberry wilt as a basis for disease risk prediction. Plant Pathol. 1996;45(1):106–14.

[3] Xiao CL, Subbarao KV. Relationships between *Verticillium dahliae* inoculum density and wilt incidence, severity, and growth of cauliflower. Phytopathology. 1998;88(10):1108–15. 10.1094/PHYTO.1998.88.10.1108 18944824

[4] Pullman GS, DeVay JE. Epidemiology of Verticillium wilt of cotton: a relationship between inoculum density and disease progression. Phytopathology. 1982;72:549–54.

[5] Ashworth JLJ, Huisman OC, Harper DM, Stromberg LK, Bassett DM. Verticillium wilt disease of cotton: Influence of inoculum density in the field. Phytopathology. 1979;69:483–9.

[6] Banno S, Saito H, Sakai H, Urushibara T, Ikeda K, Kabe T, et al Quantitative nested real-time PCR detection of *Verticillium longisporum* and *V*. *dahliae* in the soil of cabbage fields. J Gen Plant Pathol. 2011;77(5):282–91.

[7] Bilodeau GJ, Koike ST, Uribe P, Martin FN. Development of an assay for rapid detection and quantification of *Verticillium dahliae* in soil. Phytopathology. 2012;102(3):331–43. 10.1094/PHYTO-05-11-0130 22066673

[8] Debode J, Van Poucke K, Franca SC, Maes M, Hofte M, Heungens K. Detection of multiple *Verticillium* species in soil using density flotation and real-time polymerase chain reaction. Plant Dis. 2011;95(12):1571–80. 30731999 10.1094/PDIS-04-11-0267

[9] Wang YL, Wang Y, Tian CM. Quantitative detection of pathogen DNA of Verticillium wilt on smoke tree *Cotinus coggygria* . Plant Dis. 2013;97(12):1645–51. 30716826 10.1094/PDIS-04-13-0406-RE

[10] Wei F, Fan R, Dong H-T, Shang W-J, Xu X-M, Zhu H-Q, et al Threshold microsclerotial inoculum for cotton Verticillium wilt determined through wet-sieving and real-time quantitative PCR. Phytopathology. 2015;105:220–9. 10.1094/PHYTO-05-14-0139-R 25098492

[11] Johnson KB, Apple JD, Powelson ML. Spatial patterns of *Verticillium dahliae* propagules in potato field soils of Oregon Columbia Basin. Plant Dis. 1988;72(6):484–8.

[12] Smith VL, Rowe RC. Characteristics and distribution of propagules of *Verticillium dahliae* in Ohio potato field soils and assessment of 2 assay-methods. Phytopathology. 1984;74(5):553–6.

[13] Wheeler TA, Madden LV, Riedel RM, Rowe RC. Distribution and yield-loss relations of *Verticillium dahliae*, *Pratylenchus penetrans*, *P scribneri*, *P cerenatus*, and *Meloidogyne hapla* in commercial potato fields. Phytopathology. 1994;84(8):843–52.

[14] Xiao CL, Hao JJ, Subbarao KV. Spatial patterns of microsclerotia of *Verticillium dahliae* in soil and Verticillium wilt of cauliflower. Phytopathology. 1997;87(3):325–31. 18945176 10.1094/PHYTO.1997.87.3.325

[15] Evans G, Gleeson AC. An evaluation of the sampling variation when estimating the population of *Verticillium dahliae* in field soil. Ann Appl Biol. 1980;95:177–84.

[16] Johnson DA, Zhang H, Alldredge JR. Spatial pattern of Verticillium wilt in commercial mint fields. Plant Dis. 2006;90:789–97. 10.1094/PD-90-0789 30781241

[17] Navas-Cortes JA, Landa BB, Mercado-Blanco J, Trapero-Casas JL, Rodriguez-Jurado D, Jimenez-Diaz RM. Spatiotemporal analysis of spread of infections by *Verticillium dahliae* pathotypes within a high tree density olive orchard in southern Spain. Phytopathology. 2008;98(2):167–80. 10.1094/PHYTO-98-2-0167 18943193

[18] Harris DC, Yang JR, Ridout MS. The detection and estimation of *Verticillium dahliae* in naturally infested soil. Plant Pathol. 1993;42(2):238–50.

[19] Dower WJ, Miller JF, Ragsdale CW. High efficiency transformation of *E*. *coli* by high voltage electroporation. Nucleic Acids Res. 1988;16:6127–45. 3041370 10.1093/nar/16.13.6127 PMC336852

[20] Zhang WW, Jiang TF, Cui X, Qi FJ, Jian GL. Colonization in cotton plants by a green fluorescent protein labelled strain of *Verticillium dahliae* . Eur J Plant Pathol. 2013;135(4):867–76.

[21] Madden LV, Hughes G, van den Bosch F. The study of plant disease epidemics. St. Paul, Minnesota, USA: The American Phytopathological Society; 2007 421 p.

[22] Dhanasekaran S, Doherty TM, Kenneth J, Grp TBTS. Comparison of different standards for real-time PCR-based absolute quantification. J Immunol Methods. 2010;354(1–2):34–9. 10.1016/j.jim.2010.01.004 20109462

[23] Sivaganesan M, Haugland RA, Chern EC, Shanks OC. Improved strategies and optimization of calibration models for real-time PCR absolute quantification. Water Res. 2010;44(16):4726–35. 10.1016/j.watres.2010.07.066 20701947

[24] Griffiths DA. The fine structure of developing microsclerotia of *Verticillium dahliae* Kleb. Archiv Mikrobiol. 1970;74(3):207–12.

[25] Xu X-M, Ridout MS. Effects of initial conditions, sporulation rate, and spore dispersal gradient on the spatio-temporal dynamics of plant disease epidemics. Phytopathology. 1998;88(10):1000–12. 10.1094/PHYTO.1998.88.10.1000 18944811

[26] Xu X-M, Ridout MS. Effects of quadrat size and shape, initial epidemic conditions, and spore dispersal gradient on spatial statistics of plant disease epidemics. Phytopathology. 2000;90(7):738–50. 10.1094/PHYTO.2000.90.7.738 18944493

[27] Mol L, VanHalteren JM, Scholte K, Struik PC. Effects of crop species, crop cultivars and isolates of *Verticillium dahliae* on the population of microsclerotia in the soil, and consequences for crop yield. Plant Pathol. 1996;45(2):205–14.

[28] Wheeler TA, Madden LV, Rowe RC, Riedel RM. Effects of quadrat size and time of year for sampling of *Verticillium dahliae* and lesion nematodes in potato fields. Plant Dis. 2000;84(9):961–6. 10.1094/PDIS.2000.84.9.961 30832027

[29] Evans G, Wilhelm S, Snyder WC. Quantitative studies by plate counts of propagules of the Verticillium wilt fungus in cotton field soils. Phytopathology. 1967;57:1250–5.

[30] Koroleva NS, Kas'yanenko AG, MIller VR. Studies on the ecology, populations and evolution of *Verticillium* species. I. Population density dynamics of *Verticillium dahliae* Kleb. Mycology and Phytopathology. 1986;20:509–12. Russian.

[31] França SC, Spiessens K, Pollet S, Debode J, De Rooster L, Callens D, et al Population dynamics of Verticillium species in cauliflower fields: Influence of crop rotation, debris removal and ryegrass incorporation. Crop Protection.2013;54:134–41.

[32] Easton GD, Nagle ME, Bailey DL. A method of estimating *Verticillium albo-atrum* propagules in field soil and irrigation water. Phytopathology. 1969;59:1171–2.

[33] García-Cabello S, Pérez-Rodríguez M, Blanco-López MA, López-Escudero FJ. Distribution of *Verticillium dahliae* through watering systems in widely irrigated olive growing areas in Andalucia (Southern Spain). Eur J Plant Pathol. 2012;133:877–85.

