# Supplementary figures and images for: Spatial Pattern of Verticillium dahliae Microsclerotia and Cotton Plants with Wilt Symptoms in Commercial Plantations
Source: PLoS One. 2015 Jul 13;10(7):e0132812. doi: 10.1371/journal.pone.0132812 (PMC4500557; doi:10.1371/journal.pone.0132812)

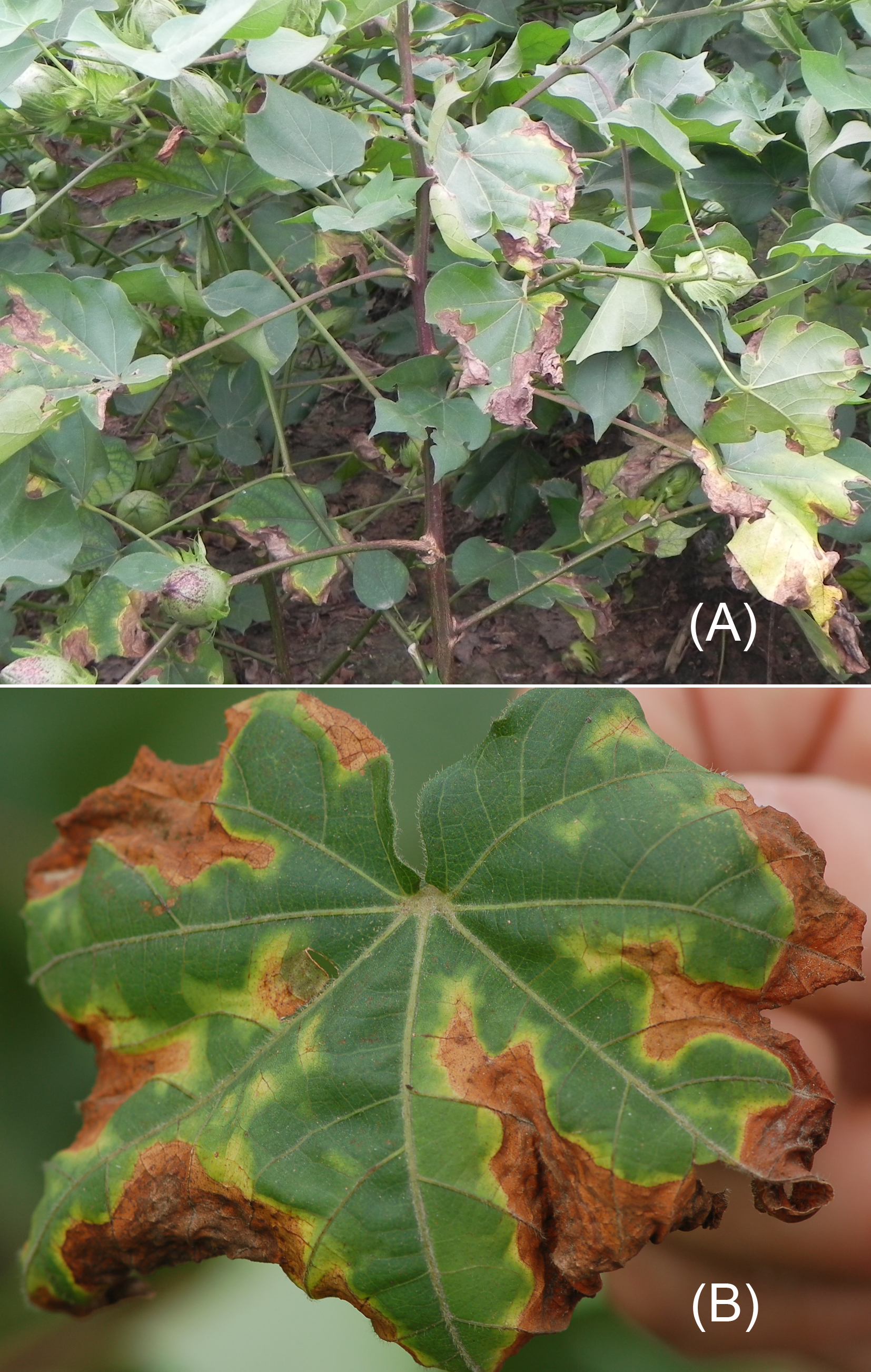

Supplement: S1 Fig — A whole cotton plant with severe wilt (A) and a cotton leaf with the typical Verticillium wilt (B). Photos were taken in a commercial cotton field. (TIF) [file pone.0132812.s001.tif]

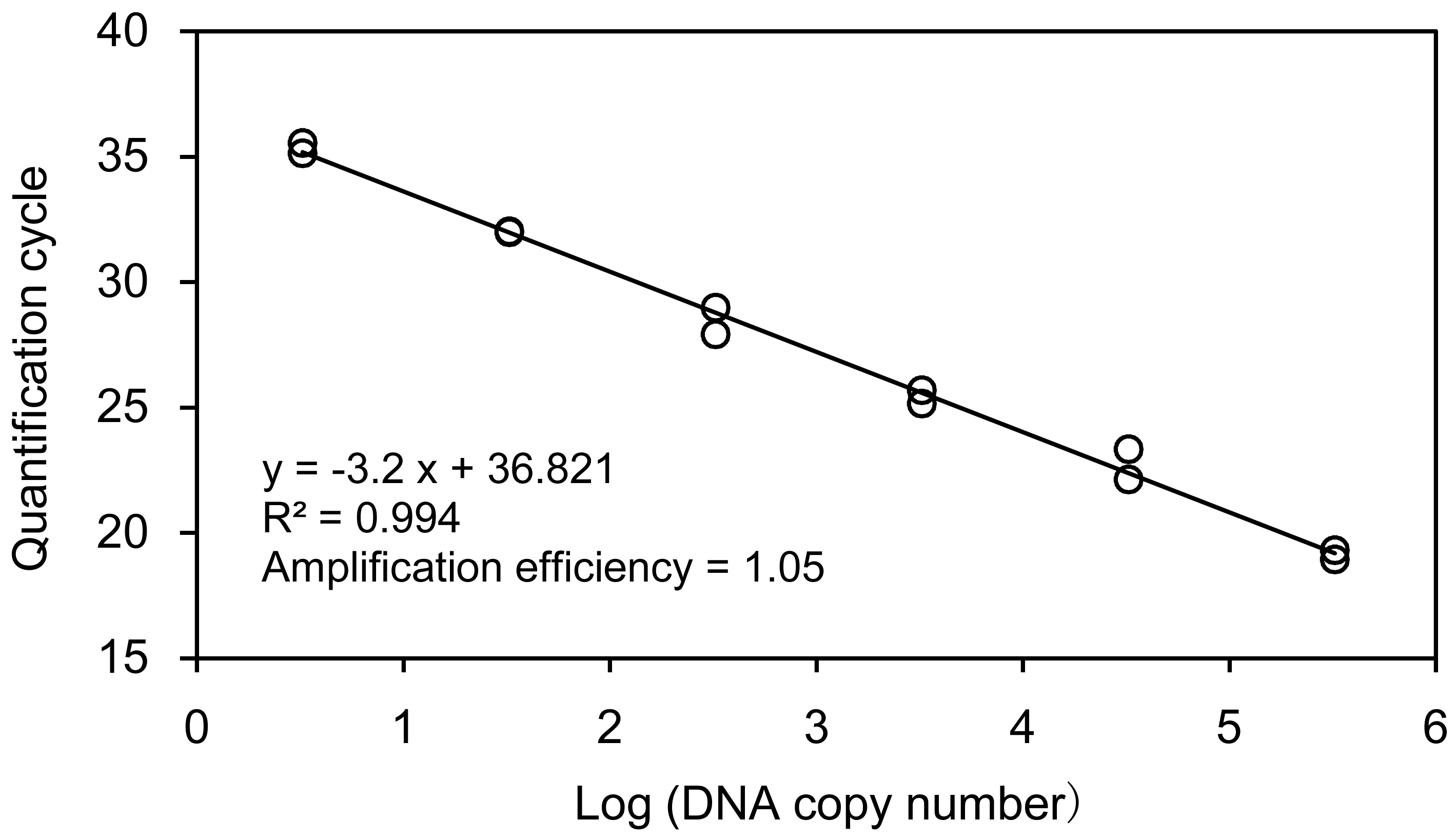

Supplement: S2 Fig — A standard curve relating the quantification cycle value to copy number (logarithmically transformed) of dilution series plamids of intergenic spacer (IGS) fragment from the JY strain. Amplification efficiency was E=10−slope−1−1. (TIF) [file pone.0132812.s002.tif]
